# Lexical Representations of the Native and Second Languages During L2 Word Reading in Chinese–English Bilinguals

**DOI:** 10.1162/NOL.a.255

**Published:** 2026-06-16

**Authors:** Xiaoyu Liu, Lala Gu, Xiaoxue Feng, Yuan Feng, Xingying Lin, Nannan Gu, Leilei Mei

**Affiliations:** Philosophy and Social Science Laboratory of Reading and Development in Children and Adolescents, South China Normal University; Ministry of Education, Guangzhou, China; School of Psychology, Zhejiang Normal University, Jinhua, China; Center for Studies of Psychological Application, South China Normal University, Guangzhou, China; School of Psychology, South China Normal University, Guangzhou, China

**Keywords:** bilingualism, EEG, fMRI, lexical access, representational similarity analysis

## Abstract

The non-selective processing hypothesis for bilinguals posits that during the processing of the target language, the lexical information of the non-target language is concurrently activated. Nonetheless, the lexical representation of bilinguals in both languages and the temporal dynamics of lexical information representation remain unclear. Here, we utilized fMRI in Experiment 1 and EEG in Experiment 2, combined with representational similarity analysis (RSA), to explore the neural representation of lexical access in both the target and non-target languages during L2 word reading and the modulatory effects of processing demands. Results of two Experiments jointly revealed that the lexical information of L2 was represented widely and earlier during L2 lexical processing, whereas the lexical information of L1 was represented widely and earlier during L2 semantic processing. These findings provide spatiotemporally integrated evidence for the bilingual non-selective access hypothesis, indicating that non-selective processing occurs only under conditions of high semantic processing demands.

## INTRODUCTION

Word reading is a crucial cognitive process in language comprehension (Fiez & Petersen, 1998; Murphy et al., 2019). This process is supported by an extensive network of brain regions that operate in a coordinated manner. The left occipitotemporal region is considered to be associated with word form recognition (Dehaene & Cohen, 2011; Li et al., 2024; McCandliss et al., 2003; Vin et al., 2024). The left frontal cortex facilitates the conversion from orthographic to phonological information (Ferreira et al., 2021; Jamal et al., 2012). Phonological processing is further supported by the left middle and posterior parts of the superior temporal cortex, the inferior frontal gyrus (IFG), and the parietal cortex (Graves et al., 2023; Murphy et al., 2019). Additionally, the superior temporal gyrus (STG), middle temporal gyrus (MTG), and angular gyrus (AG) have been reported to play a vital role in semantic processing (Li et al., 2023; Timofeeva et al., 2024).

Similar to monolinguals, bilinguals recruit the key brain regions described above for word reading (Marks et al., 2023; Zhan et al., 2023). However, compared with monolinguals who only master one language, the language processing of bilinguals is more complex (Fu et al., 2017; Kroll & Dijkstra, 2010). Specifically, the bilingual selective access hypothesis posits that when bilinguals process lexical information in one language, the lexical information of the other language is not activated or represented (Dussias, 2003; Olson, 2017; Philipp & Huestegge, 2015). In contrast, an increasing number of researchers have advocated for the bilingual non-selective access hypothesis in recent years (de Groot et al., 2000; Duyck & Warlop, 2009; Lauro & Schwartz, 2017; Liu et al., 2023, 2024), which posits that when bilinguals read words in a language, words in the other language are also activated and represented in the brain (Dijkstra et al., 2000, 2018; van Hell & Dijkstra, 2002). This hypothesis is supported by evidence from the cognate facilitation effect (Lemhöfer et al., 2008; Schwartz et al., 2007; Voga & Granger, 2007), the homographs inhibitory effect (de Groot et al., 2000), and the (masked) priming effect of translation equivalents across languages (Chen et al., 2014; Xia & Andrews, 2015). These results indicate that bilinguals activate both language systems simultaneously when they process words in one language.

Based on extensive evidence for bilingual non-selective access, Dijkstra and van Heuven (2002) proposed the Bilingual Interactive Activation (BIA) + model, which is optimized based on the BIA model (Dijkstra et al., 1999). The model posits that bilinguals’ two languages are stored in an integrated mental lexicon, and the visual input will simultaneously activate all matching lexical candidates in both L1 and L2 from the early processing stage and inhibit the letters without them, which means the lexical access of both languages is non-selective. When bilinguals process a string of letters, visually presented information specific to the location of each letter activates letters with these features while inhibiting letters without them. Additionally, the BIA+ model distinguishes between the lexical recognition system and the task decision system. It posits that the lexical recognition system is directly influenced only by linguistic factors such as lexical, semantic, and syntactic information. The task decision system is affected by non-linguistic factors such as processing demands, external contexts, and personal experiences. This system does not directly impact the information processing of the lexical recognition system itself but only modifies the output of the lexical recognition system, which affects the final behavioral response under different processing demands (Dijkstra et al., 1999).

Although a large amount of research has been conducted on the lexical access of bilinguals in both languages (Chen et al., 2014; Wang et al., 2020; Xia & Andrews, 2015), there are at least three limitations. First, previous research mainly explored bilingual lexical access by an unimodal approach, such as behavioral research (e.g., cross-linguistic priming paradigms and cognate judgment tasks) (Chen et al., 2014; Lemhöfer et al., 2008; Schwartz et al., 2007; Voga & Granger, 2007; Xia & Andrews, 2015), electroencephalography (EEG) research (Midgley et al., 2008; Schwartz et al., 2007; Thierry & Wu, 2007), and fMRI research (Hu et al., 2023; Roos et al., 2023). However, such studies can only provide evidence for the BIA+ model from separate temporal or spatial dimensions, which offers limited insight into a unified account of when processing unfolds and what cognitive components are involved. The spatiotemporal dynamics of lexical information processing of two languages in bilinguals remain unclear. Therefore, multimodal neuroimaging methods are essential in a single study to comprehensively investigate the neural representation of lexical access in bilinguals from a spatiotemporally integrated perspective.

Second, previous studies mainly adopted the univariate analysis approach (i.e., the amplitude analysis in EEG research and the activation analysis in fMRI research) to explore the neural activities of the two languages in bilinguals (DeLuca et al., 2020; Jiao et al., 2020; Kuper et al., 2021). This univariate analysis approach simply averages the neural signals, which lose the fine-grained pattern information (Gu et al., 2023; Lu et al., 2021; Wang et al., 2012). In contrast, the multivariate approach that associates the distributed neural activity patterns with the lexical information can provide a more comprehensive insight into the lexical information representation. Consequently, the multivariate method (e.g., representational similarity analysis) is needed to explore the spatiotemporal representation of lexical information in both L1 and L2 of bilinguals from a quantitative perspective.

Third, the BIA+ model posited that the task decision system affects the final behavioral response under different processing demands, rather than the lexical identification system. In other words, the individual response differences across different processing demands are not attributed to lexical identification in the early stage but rather to the modulation of the subsequent task schema system on input linguistic information. However, a number of studies have found that processing demands affect the cognitive and neural mechanisms of word reading (Branzi et al., 2022; Price & Devlin, 2011). There is also evidence of the modulatory effects of processing demands on lexical memory and brain activations of the two languages in bilinguals (Chen et al., 2014; Dimitropoulou et al., 2011; Li et al., 2022; McPhedran & Lupker, 2021; Voga & Granger, 2007). Nevertheless, it remains unclear whether and how processing demands modulate the neural activity of the two languages in bilingual individuals, as well as the non-selective processing mechanism in bilinguals. Therefore, it is essential to adopt multiple reading tasks in one study to investigate the modeling assumptions directly.

To overcome the above three limitations, the present study used two typical reading tasks (i.e., the lexical decision task with high processing demands for lexical access and the semantic judgment task with high processing demands for semantic processing) in combination with the high spatial-resolution fMRI (Experiment 1) and the high temporal-resolution EEG (Experiment 2) to investigate the lexical information representations in L1 and L2 of Chinese–English bilinguals and the modulatory effects of processing demands. To minimize the interaction between the two languages, participants were required to complete only tasks for the English (L2) lexical material, because it is more likely to observe the co-activation of the two languages in the L2 processing of unbalanced bilinguals. In addition, to directly exploring the lexical information representation of the two languages, we manipulated the degree of variability between the orthographic and semantic information of English words and their translation words (i.e., Chinese words) for use in subsequent representational similarity analyses (RSA). RSA can explore the neural representation of stimulus features (e.g., English phonology or Chinese orthography) by calculating the correlation between the neural matrix and the stimulus feature matrix (Kriegeskorte et al., 2008; Liu et al., 2024; Nili et al., 2014), which provides a new perspective for addressing bilingual lexical information representation and access.

## METHODS

### Experiment 1

Using two typical reading tasks (i.e., the lexical decision task and the semantic judgment task) and RSA, Experiment 1 aimed to explore the neural representation of lexical information in bilinguals’ two languages during L2 word reading, as well as the modulatory effects of processing demands. Drawing on the bilingual non-selective view (Liu et al., 2023; van Hell & Dijkstra, 2002), we hypothesized that lexical information of both languages would be represented when bilinguals read L2 words, and processing demands would modulate the neural representations of lexical information in both languages of bilinguals.

#### Participants

Twenty-four unbalanced Chinese–English bilinguals (14 females, mean age = 21.04, *SD* = 1.744, range 19–24) were recruited for Experiment 1. They all had more than 10 years of L2 learning experience (mean = 13.58 ± 1.91 years). To assess participants’ English proficiency in more detail, we further measured their English proficiency by a subjective 7-point self-rating scale (1 = minimal proficiency, 7 = native-like proficiency) and an objective LexTALE test (Lemhöfer & Broersma, 2012). Results showed that the mean score of self-rated English proficiency was 3.70 (*SD* = 0.08), and the mean score of the LexTALE test was 56.57 (*SD* = 6.64). All participants had normal vision and were right-handed (Snyder & Harris, 1993). They signed an informed consent form before the experiment. Institutional Review Board has approved the ethical procedures.

#### Materials

The experimental materials consisted of 30 English words, 90 English pseudowords, and 10 color words. Specifically, the 30 English words were selected from the English Lexicon Project (Balota et al., 2007) and belonged to medium-high frequency words (mean = 9.45/million). To evaluate the materials, another 20 participants were recruited and divided into two groups (10 participants each). One group was asked to translate the 30 English words into Chinese. The translation consistency of all the English words was above 70% (mean = 8.83, *SD* = 1.186). The word frequency of these Chinese words (mean = 2.97/million) is medium-high (Cai & Brysbaert, 2010). The other group was asked to rate the familiarity of the 30 English words on a scale of 1–7 (1 = very unfamiliar and 7 = very familiar), and the familiarity of all the English words was 6.50 ± 0.529. The result indicates a high level of familiarity with English words.

In addition, 90 English pseudowords generated by the English Pseudowords Generation Software (https://crr.ugent.be/programs-data/wuggy) were used as the probe stimuli for the lexical decision task. All the English pseudowords were matched with real English words in the dimensions of subsyllabic segments and letter length. The 90 English pseudowords were assigned to six runs, with 15 English pseudowords in each run. The letter length and subsyllabic segments of these pseudowords were also matched across different runs. The materials were produced as black pictures on a gray background.

Ten common color words were used as the probe stimuli for the semantic judgment task. 10 words were divided into two sets (five words per set). In each run, all words from one set were used to generate five probe stimuli. The two sets of color words did not differ in word length (U = 11.5, *p* = 0.827) or word frequency (U = 12, *p* = 0.917).

#### Procedure

**Figure 1.**
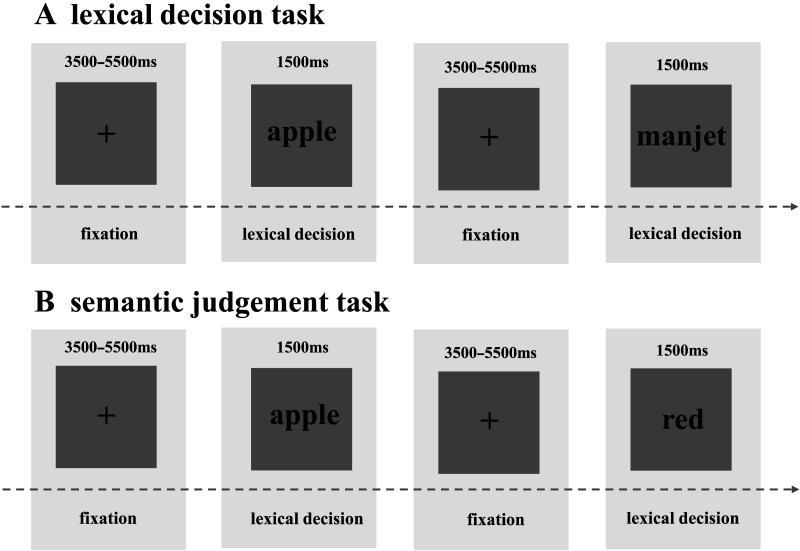
Procedures of fMRI tasks. (A) In the lexical decision task, participants judged whether the letter string was a real word or not. (B) In the semantic judgment task, participants were instructed to press a key once a color word appeared.

Participants performed the lexical decision task and the semantic judgment task during fMRI (Figure 1). The order of the two tasks was counterbalanced across participants. Both tasks used event-related designs and consisted of six runs. All experimental materials were presented in a pseudo-random sequence programmed by a visual display programming toolbox (Psychtoolbox-3) in MATLAB 2013b.

In the lexical decision task, each run consisted of 45 trials, with 30 trials of English words and 15 trials of English pseudowords. In each trial, a word or pseudoword (i.e., stimulus) was displayed for 1.5 s, followed by a blank interval (mean = 4.5 s, ranging from 3.5 s to 5.5 s) presented as jitter between stimuli. Meanwhile, participants were instructed to determine whether the letter string on the screen was a real word (by pressing the button “1”) or not (by pressing the button “4”). The response mappings were counterbalanced between participants. This task lasted 27 minutes.

In the semantic judgment task, each run consisted of 35 trials with 30 English words and 5 English color words. In each trial, the word was presented for 1.5 s, followed by a blank interval (mean = 4.5 s, ranging from 3.5 s to 5.5 s) presented as jitter between words. Participants were instructed to view the words carefully and press “1” using their left index or “4” using their right index once a color word appeared (the button was counterbalanced between participants). This task lasted 21 minutes.

#### MRI data acquisition

All image data were acquired using a 3.0 T Siemens Prisma MRI scanner at South China Normal University. Structural images were scanned using a T1-Weighted three-dimensional fast gradient echo pulse sequence in sagittal section (TR/TE = 2,530/1.94 ms, TI = 1,100 ms, Flip = 7°, FOV = 256 mm × 256 mm, pixel matrix = 256 × 256, voxel size = 0.5 × 0.5 × 1.0 mm (McCandliss et al., 2003). The number of scanned layers was 176.

Functional imaging was performed using a T2*-weighted multiple excitation gradient echo planar imaging sequence with TR/TE = 2,000/30 ms, FOV = 224 mm × 224 mm, Flip = 90°, and voxel size = 2.0 × 2.0 × 2.0 mm (McCandliss et al., 2003). The scans were taken in horizontal sections, with a total of 58 layers scanned at 3-mm intervals, covering the whole brain and part of the cerebellum.

#### Activation analysis

Image preprocessing was performed by FEAT v6.0 in FSL (FMRIB’s Software Library). To ensure that the acquired MRI signal was stable, the first three volumes of each scan sequence were automatically deleted. Next, the remaining images were subjected to slice timing and head motion linear correction. All images were then spatially smoothed using a 5 mm full-height half-width Gaussian kernel and high-pass filtered in the 100 s time cutoff. All scanning sequences of all participants did not exceed 2 mm in any one head movement amount, and the head movement deflection angle was less than 2°. Finally, data registration was performed by aligning each participant’s functional image data to the respective brain structural space and by aligning the participant’s structural images to the MNI template in standard space (Jenkinson et al., 2012).

In a first-level analysis, the experiment used a general linear model (GLM) to model the data for 30 English words of the lexical decision task or the semantic judgment task for each participant and each run. The probe stimuli (i.e., English pseudowords in the lexical decision task and the color words in the semantic judgment task) were excluded in the subsequent analysis. Specifically, the onset and duration of the predictor variables (EVs) were convolved with a standard Double-Gamma HRF. For each predictor variable, six head-movement parameters were entered into the model as the nuisance event. In addition, the fixation was used as a baseline for each predictor variable to improve statistical sensitivity.

In a second-level analysis, the data were concentrated across the six runs using a fixed-effect model for each participant and each task. Finally, group activation images were obtained using a random-effects model to ensure the generalizability of the results to the group level. All group-level results were corrected by a cluster probability of *p* < 0.05 and thresholded at *Z* > 2.6 (Jezzard et al., 2001).

#### Rating-based dissimilarity matrices

After scanning, participants were instructed to rate the orthographic/phonological similarity of the 30 English words and Chinese equivalent words (30 × (30 − 1) / 2 = 435 pairs for each language). The rating was performed on a 7-point scale, with one being completely different and seven being completely the same. For the phonological similarity evaluation of English words, participants were instructed to evaluate the similarity between two words in terms of the number of common phonemes and the location of the phonemes. For the orthographic similarity evaluation of English words, participants were asked to evaluate the degree of similarity between two words in terms of the length of the words, the number of identical letters, and the position of identical letters. For the phonological similarity evaluation of Chinese words, participants were asked to consider the degree of similarity between the initial and final characters in terms of consonants, rhymes, and lexical tones. To evaluate the orthographic similarity of Chinese words, participants were asked to consider the degree of similarity between the initial and final characters in terms of structure and components. To ensure that participants understood the rules for evaluating the above materials, examples of evaluations ranging from “completely different” to “completely the same” were provided before formal evaluation.

Based on ratings of the above material information, the representational dissimilarity matrix (RDM) of orthographic/phonological information of English and Chinese words was constructed separately. Specifically, the ratings of all participants were averaged to obtain similarity ratings based on English orthography, English phonology, Chinese orthography, and Chinese phonology. The rating scores were reversed (i.e., 8 minus the rating score) to obtain the dissimilarity matrices (DSMs). The DSMs of orthography and phonology were not correlated between the two languages (orthography: *r* = 0.01, *p* = 0.821; phonology: *r* = 0.06, *p* = 0.184).

#### Representational similarity analysis

To investigate the lexical information representation (i.e., orthographic and phonological information) in L1 and L2 during L2 word reading, we conducted region of interest (ROI)-based RSA and whole-brain searchlight RSA. Specifically, consistent with previous studies (Liu et al., 2024; Qu et al., 2022), the unsmoothed functional data were used to re-estimate the GLM for each run and each participant. Each of the 30 experimental stimuli (all real and non-color words) was used as a separate regression variable to obtain the activity pattern of each word.

**Figure 2.**
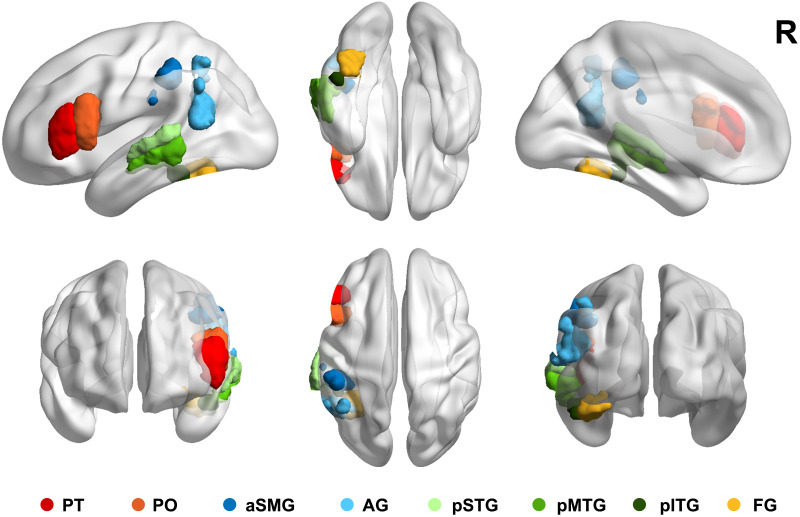
Spatial localization map of the regions of interest (ROIs). All ROIs were identified based on the Harvard-Oxford atlas (Maximum probability threshold: 25%), and then overlapped onto the brain map derived from a meta-analysis of 521 reading-related studies from the Neurosynth. PT = pars triangularis, PO = pars opercularis, aSMG = anterior superior marginal gyrus, AG = angular gyrus, pSTG = posterior superior temporal gyrus, pMTG = posterior middle temporal gyrus, pITG = posterior inferior temporal gyrus, and FG = fusiform gyrus. R = right.

In the ROI-based RSA, we only defined left brain regions as ROIs for two reasons. First, previous studies have consistently identified the left hemisphere as a crucial region in word reading (Gu et al., 2024; Klaus & Hartwigsen, 2019; Zuk et al., 2018). Second, findings from the meta-analysis of 521 reading-related studies further corroborated that the right hemisphere exhibits weaker activation patterns in reading processing. Specifically, based on the key brain regions of word reading reported in previous studies (Binder et al., 2009; Gu et al., 2026; Liu et al., 2024; Murphy et al., 2019; Timofeeva et al., 2024; Turker et al., 2023), combined with the activation results in the lexical decision and the semantic judgment task in the present study, we defined eight ROIs. Including the ventral (i.e., pars triangularis, PT) and dorsal (i.e., pars opercularis, PO) subregions of the left IFG, the anterior superior marginal gyrus (aSMG), the angular gyrus (AG), the posterior superior temporal gyrus (pSTG), the posterior middle temporal gyrus (pMTG), the posterior inferior temporal gyrus (pITG), and the fusiform gyrus (FG). All above ROIs were identified based on the Harvard-Oxford probabilistic atlas with a maximum probability threshold of 25% within the FSL software (Supporting Information Table S1; Supporting Information can be found at https://doi.org/10.1162/NOL.a.255). In addition, to more precisely localize brain regions related to word reading, the structural ROIs defined above were overlapped with the brain map based on a meta-analysis of 521 word-reading-related studies from the Neurosynth (https://www.neurosynth.org/) to obtain the final ROIs (Figure 2). Subsequently, for each task and each run, the activity pattern of each ROI was estimated for each word. The dissimilarity value of any pair of words was computed (1 minus Pearson’s correlation) and entered into the RDM. For each task, the RDMs of six runs were averaged to obtain the final neural RDM.

A 5,000 times permutation test was used to explore the neural representation of orthographic and phonological information of L1/L2 for each ROI. Specifically, Spearman’s correlation between the DSM of lexical information and the neural RDM was performed as the actual observed value of representation in the region. Subsequently, the DSM of lexical information was randomly disrupted and then correlated with the neural RDM as a random sample. All correlation coefficients were Fisher-*Z* transformed. The *p* value of the actual observation was computed by (1 + the number of permutated *Z* values greater than the actual *Z*)/5001 (Nili et al., 2014). The actual observation was considered statistically significant when it was greater than 95% in the null distribution. Ultimately, the false-positive rate (FDR) correction was used to control for multiple comparisons.

In the whole-brain searchlight RSA, a 5 × 5 × 5 cube (125 voxels) was identified based on the center voxel to perform whole-brain searchlight analysis for each participant and each run following previous studies (Kim et al., 2020; Liu et al., 2024). This searchlight covered all voxels of the whole brain. Then, activation patterns for all words were extracted based on each cube. The DSM of the activation pattern was calculated by subtracting Pearson’s correlation value from one and transformed into Fisher’s *Z*-scores. Six repetitions in each run were averaged to obtain the neural DSM. Subsequently, the correlations between the neural DSM and the DSMs of the four lexical components (i.e., English orthography, English phonology, Chinese orthography, and Chinese phonology) were computed using the Spearman correlation, respectively. Finally, all the correlation coefficients were converted to Fisher-*Z* scores and mapped to the center voxel of each cube to obtain a *Z*-value brain map (*Z* map). The lexical information (i.e., English orthography, English phonology, Chinese orthography, and Chinese phonology) representation maps were subjected to a group-level random-effects model using permutation-based nonparametric statistics in SnPM (https://warwick.ac.uk/fac/sci/statistics/staff/academic-research/nichols/software/snpm8/). The permutation was repeated 10,000 times, and a 95% confidence interval was used to account for multiple comparisons across voxels (Liu et al., 2024).

### Experiment 2

Using EEG and RSA, Experiment 2 aimed to further explore the temporal dynamics of lexical access of L1 and L2 during L2 word reading. Based on previous findings, we expected that lexical information of both L1 and L2 would be represented during L2 word reading, and processing demands would modulate the neural representation of lexical information in the lexical recognition stage.

#### Participants

Twenty-seven unbalanced Chinese–English bilinguals (17 females, aged from 18 to 25 years old, mean age = 21.6 ± 2.2 years) were recruited for Experiment 2 with more than 10 years of L2 learning experience (mean = 12.8 ± 2.4 years). The mean score of self-rated English proficiency was 3.59 (*SD* = 0.08), and the mean score of the LexTALE test was 59.68 (*SD* = 6.79). All participants had normal or corrected-to-normal vision and were right-handed (Snyder & Harris, 1993). They signed an informed consent form before the experiment. Institutional Review Board has approved the ethical procedures.

#### Materials

The materials in Experiment 2 were consistent with Experiment 1.

#### Procedure

The experiment was conducted in a soundproof and electrically shielded room by E-prime 2.0. The tasks in Experiment 2 were consistent with Experiment 1, which included six runs of the lexical decision task (lasting 27 min) and six runs of the semantic judgment task (lasting 21 min). The keys for “1” and “4” in the fMRI scans have been reassigned to the “F” and “J” keys on the keypad. The order of the two tasks and the hands used for pressing the keys were balanced across participants. Before the formal experiment, there was a practice for participants to understand the procedure of the entire experiment.

#### Electroencephalogram recordings and data analysis

The EEG and electrooculogram (EOG) data were recorded by a Neuroscan SynAmps2 amplifier with a Neuroscan 64-channel QuickCap. The EOG data were recorded from horizontal electrodes at the outer canthus of both eyes and vertical electrodes in the upper and lower orbits of the left eye to detect the ocular artifacts. The online reference was located between Cz and CPz, and the sampling rate was 1000 Hz. Most electrode resistances were kept below 5 kΩ, and a few electrode resistances were below 10 kΩ (1.06% per participant on average).

The electrophysiological signals were analyzed using MATLAB R2018b and EEGLAB v2022.1. The signals were re-referenced offline by averaging bilateral mastoid electrodes and were filtered with a band-pass filter of 0.1–30 Hz. An independent component analysis procedure was conducted to identify and remove the electrooculographic artifacts and muscle activity (Jung et al., 2001). The EEGs were then segmented into 1,000 ms epochs, with 200 ms before and 800 ms after the word onset, and were baseline corrected by subtracting the mean pre-stimulus signal (Xu et al., 2023). Any epochs with artifacts exceeding ±100 *μ*V were removed. Finally, the EEG epochs were averaged across six runs for each task and each participant to obtain the event-related potential (ERP) responses in the two tasks.

To investigate whether the classical ERP components were evoked in two tasks, two electrodes in the bilateral occipitotemporal cortex (PO7 and PO8) and four electrodes in the left frontal and parietal sites (FC1, FC3, C1, C3) were selected to detect the N170, P200, and N400 components based on previous studies (Barber et al., 2013; Mahé et al., 2015; Simon et al., 2004).

#### Spatiotemporal RSA

Spatiotemporal RSA was conducted to explore the lexical information access of both languages during the L2 lexical decision task and semantic judgment task (Lu et al., 2015; Salmela et al., 2018).

First, we extracted the mean ERP amplitude for each participant to construct the neural RDM. Specifically, we merged the electrodes into nine subregions to improve the signal-to-noise ratio of the signal based on previous studies (Sun et al., 2025; Zhang et al., 2021). Notably, due to the fundamental differences in the neurophysiological signals and spatial/temporal resolution between fMRI and ERP techniques, these nine subregions in Experiment 2 do not correspond to the ROIs identified in Experiment 1. The neural activity of each subregion was the mean activity of all electrodes within the subregion (the information of nine subregions was detailed in Supporting Information Table S2). The temporal features were selected from 200 ms pre-stimulus to 650 ms post-stimulus of epochs, utilizing a 100 ms sliding window with a step size of 1 ms. In each subregion, a data vector was extracted from the 100 time points to represent the spatiotemporal pattern of ERP activity of the middle time point. Ultimately, the neural RDM was constructed using the value of 1 minus the Pearson correlation between all epochs in each subregion and at each time point.

Then, we constructed four DSMs of lexical variables (i.e., orthographic and phonological DSMs of L1 and L2) consistent with Experiment 1 by using the rating results from Experiment 2.

Finally, the neural RDM was correlated with the above four DSMs by Spearman correlation at each time point and subregion to investigate the spatiotemporal representational patterns of orthographic and phonological information in L1 (Chinese) and L2 (English). These correlation coefficients were then transformed into Fisher’s Z-scores for further analysis (Fischer-Baum et al., 2017). The significance was estimated by a cluster-based non-parametric permutation test by the FieldTrip toolbox (https://fieldtrip.fcdonders.nl/) to avoid inflated false positive rates from multiple comparisons (Oostenveld et al., 2011; Xu et al., 2023). Specifically, the statistical analyses were conducted for each time point. Significant time points (*p* < 0.05) were selected and clustered into connected sets based on temporal adjacency. The statistical values of all time points within each cluster were summed to obtain cluster-level statistics. The null hypothesis corresponds to a correlation coefficient of 0. The permutation was repeated 5,000 times in each Spearman correlation, and a 95% confidence interval was applied. Clusters were considered significant if they remained significant (*p* < 0.05) for at least 15 ms (Li et al., 2022; Zhao et al., 2017).

## RESULTS

### Experiment 1

#### Behavior results

For the lexical decision task, the accuracy was 0.98 (*SD* = 0.025) and the response time was 588.43 ms (*SD* = 179.47). For the semantic judgment task, the accuracy was 0.996 (*SD* = 0.006) and the response time was 503.92 ms (*SD* = 124.08). These behavioral results indicate that participants performed the experimental task carefully in the MRI.

#### Brain activation of L2 word reading

**Figure 3.**
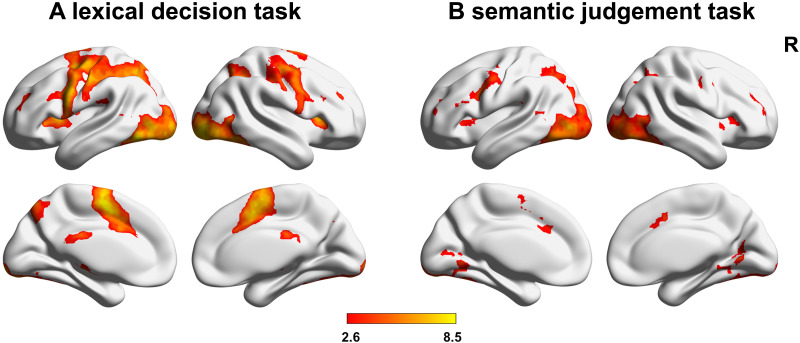
Brain activation results. (A) Brain activation for the lexical decision task. (B) Brain activation for the semantic judgment task. All activations were thresholded at *Z* > 2.6 (whole-brain corrected). R = right.

Brain activation results showed that both the lexical decision and the semantic judgment tasks evoked neural activity in classic brain networks associated with word reading (Jung & Lambon Ralph, 2023; Kuper et al., 2021; Wong et al., 2020), including the bilateral prefrontal cortex, precentral gyrus, postcentral gyrus, the occipitotemporal cortex, and the occipitoparietal cortex (Figures 3A and 3B).

#### Neural representations of orthographic and phonological information of L1 and L2

**Figure 4.**
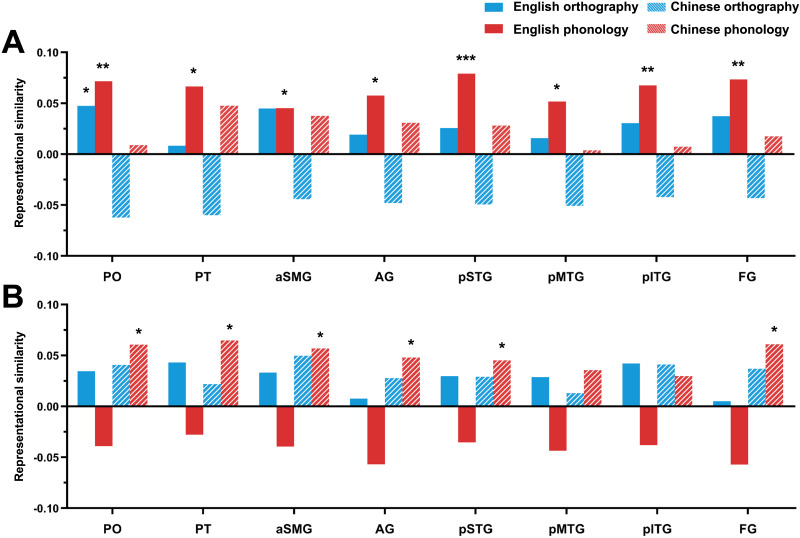
ROI-based RSA results. (A) Representation similarity of lexical information in the lexical decision task. (B) Representation similarity of lexical information in the semantic judgment task. Bar plots indicate the correlations between the DSM of lexical information and the neural RDM. The *y* axis represents Spearman’s *r* value after Fisher’s *Z* transform. PO = pars opercularis, PT = pars triangularis, aSMG = anterior superior marginal gyrus, AG = angular gyrus, pSTG = posterior superior temporal gyrus, pMTG = posterior middle temporal gyrus, pITG = posterior inferior temporal gyrus, and FG = fusiform gyrus. ***: *q* < 0.001, **: *q* < 0.01, *: *q* < 0.05.

To investigate the neural representation of orthographic and phonological information of both languages during L2 word reading, we conducted ROI-based RSA in eight ROIs, including left PT, PO, aSMG, AG, pSTG, pMTG, pITG, and FG. In the lexical decision task, we observed robust neural representations for L2 lexical information in those reading-related ROIs, but relatively weak representations for L1 lexical information. Specifically, orthographic information of L2 was represented in PO (*r* = 0.05, *p* = 0.022, *q*_FDR_ = .045), aSMG (*r* = 0.04, *p* = 0.032, *q*_FDR_ = .051), and pITG (*r* = 0.03, *p* = 0.042, *q*_FDR_ = .061), while phonological information of L2 was represented in PO (*r* = 0.07, *p* = 0.001, *q*_FDR_ = .006), PT (*r* = 0.07, *p* = 0.003, *q*_FDR_ = .01), aSMG (*r* = 0.05, *p* = 0.028, *q*_FDR_ = .05), AG (*r* = 0.06, *p* = 0. 007, *q*_FDR_ = .019), pSTG (*r* = 0.08, *p* < 0.0001, *q*_FDR_ < .0001), pMTG (*r* = 0.05, *p* = 0.016, *q*_FDR_ = .037), pITG (*r* = 0.07, *p* = 0.002, *q*_FDR_ = .006), and FG (*r* = 0.07, *p* = 0.001, *q*_FDR_ = .005) (Figure 4A). For lexical information in L1, only the left PT showed phonological representation, but it was not significant after correction. (*r* = 0.05, *p* = 0.024, *q*_FDR_ = .19) (Figure 4A).

In contrast, robust lexical representations of L1 but not L2 were found in the semantic judgment task. Specifically, orthographic information of L1 was represented in the PO (*r* = 0.04, *p* = 0.047, *q*_FDR_ = .083), aSMG (*r* = 0.05, *p* = 0.018, *q*_FDR_ = .059), and the pITG (*r* = 0.04, *p* = 0.045, *q*_FDR_ = .083) (Figure 4B). Phonological representations of L1 were detected in PO (*r* = 0.06, *p* = 0.006, *q*_FDR_ = .017), PT (*r* = 0.07, *p* = 0.004, *q*_FDR_ = .017), aSMG (*r* = 0.06, *p* = 0.011, *q*_FDR_ = .021), AG (*r* = 0.05, *p* = 0.026, *q*_FDR_ = .042), pSTG (*r* = 0.05, *p* = 0.034, *q*_FDR_ = .045), FG (*r* = 0.06, *p* = 0.006, *q*_FDR_ = .017) (Figure 4B). For lexical information of L2, only relatively weak orthographic representations were observed in the PT (*r* = 0.04, *p* = 0.04, *q*_FDR_ = .152) and pITG (*r* = 0.04, *p* = 0.043, *q*_FDR_ = .152), they did not reach significance after correction (Figure 4B).

**Figure 5.**
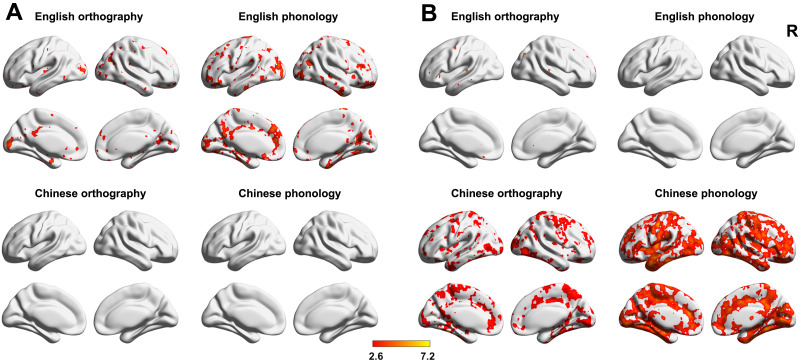
Whole-brain RSA results. (A) Whole-brain representation of lexical information in the lexical decision. (B) Whole-brain representation of lexical information in the semantic judgment task. All activations were thresholded at *Z* > 2.6 (whole-brain corrected). R = right.

The above results were further confirmed by the whole-brain searchlight RSA. For the lexical decision task, we found robust neural representations for L2 lexical information and weak neural representations for L1 lexical information. Specifically, the orthographic information of L2 was represented in the bilateral frontal pole, precentral and postcentral gyrus, SMG, MTG, pITG, the right AG, FG, and occipital cortex (Figure 5A). Phonological information of L2 was represented in a wider brain network involving bilateral SFG, MFG, IFG, precentral and postcentral gyrus, the middle and posterior temporal lobe, inferior parietal lobule (IPL), AG, and occipital cortex (Figure 5A). However, the representation of orthographic and phonological information of L1 was not detected.

For the semantic judgment task, similar to ROI-based RSA, the whole-brain searchlight RSA showed robust lexical representations of L1 and weak lexical representations of L2. Specifically, the orthographic information of L1 was represented in the bilateral SFG, MFG, precentral and postcentral gyrus, pSTG, ITG, and the temporo-occipital cortex, whereas the phonological information of L1 was more broadly represented in the bilateral SFG, MFG, IFG, precentral and postcentral gyrus, anterior and posterior temporal lobe, parietal lobe, and temporo-occipital cortex (Figure 5B). For lexical information of L2, only relatively weak orthographic representations were observed in the left IFG, precentral gyrus, the right IPL, the bilateral mSTG, and the temporo-occipital cortex. These results of both ROI-based and whole-brain searchlight RSA jointly indicate that the lexical information in L1 is activated when bilinguals read an L2 word. In addition, the processing demands modulated the neural representation of lexical information in both languages.

Taking advantage of the fMRI and RSA, Experiment 1 explored the neural representation of lexical information in two languages and the modulatory effects of processing demands during word reading in L2. Activation results showed extended activations in reading-related regions during L2 reading. Specifically, the temporo-occipital cortex has been reported to process lexical visual orthographic information (Dehaene & Cohen, 2011; Gu et al., 2024), while the PO, parietal cortex, and precentral gyrus are more involved in phonological processing (Liu et al., 2021; Skeide & Friederici, 2016), and the PT is more involved in semantic extraction and integration (Costa et al., 2006; Li et al., 2023). These findings indicated that participants were attentive during the fMRI scan. More importantly, both ROI-based and searchlight-based RSA indicated that the lexical information of both languages is concurrently activated in the brain for Chinese–English bilinguals during L2 word reading. This finding directly supports the hypothesis of bilingual non-selective access (Dijkstra et al., 1999, 2000; van Hell & Dijkstra, 2002). In addition, we found that the processing demands modulate the neural representation of lexical information in both languages. The lexical information of L2 is more clearly represented when bilinguals complete a task that focuses on the lexical information of L2, while the lexical information of L1 is more clearly represented when bilinguals complete a task that focuses more on the semantic information in L2.

Nonetheless, due to the limitations of MRI in terms of temporal resolution, the temporal dynamics of lexical information representation in bilinguals’ two languages remain unclear. Furthermore, whether the processing demands influence the early lexical recognition system or only the later task decision system is still unknown (Dijkstra & van Heuven, 1998, 2002). Consequently, Experiment 2 used high temporal resolution EEG techniques and RSA to explore the time course of lexical information processing of both languages during L2 word reading.

### Experiment 2

#### Behavioral results

For the lexical decision task, the accuracy was 0.98 (*SD* = 0.027) and the response time was 524.21 ms (*SD* = 170.16). For the semantic judgment task, the accuracy was 0.99 (*SD* = 0.027) and the response time was 461.33 ms (*SD* = 141.48). These results suggest that participants attended to the experimental task during scanning.

#### ERP waveform results

Consistent with previous studies (Barber et al., 2013; Mahé et al., 2015; Simon et al., 2004; Zhang et al., 2009), both the lexical decision and the semantic judgment tasks evoked classical ERP components, including the N170 in the bilateral occipital lobes, P200 and N400 in the left frontal and parietal lobes (Supporting Information Figure S1). These results demonstrated the reliability of the EEG data, providing a necessary foundation for the subsequent spatiotemporal RSA.

#### Spatiotemporal RSA results

**Figure 6.**
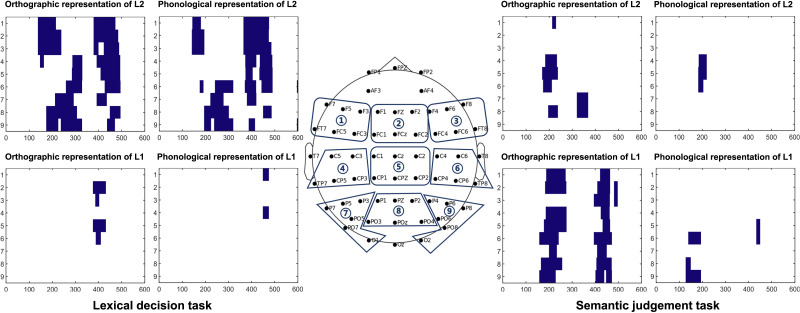
Spatial distribution of 9 subregions and the spatiotemporal RSA results in the lexical decision (the left panel) and the semantic judgement (the right panel) task. The *x* axis displays the post-stimulus time course (0–600 ms). The *y* axis indicates the 9 subregions. Blue clusters indicate significant time points using the cluster-corrected sign permutation test.

To explore the spatiotemporal representation of orthographic and phonological information of both languages during L2 word reading, we conducted RSA between the neural DSMs and four lexical variables (i.e., orthographic and phonological information of L1 and L2) DSMs for both tasks (Figure 6). In the lexical decision task, the lexical information of L2 was represented extensively throughout the whole brain, but the representations for L1 lexical information were relatively weak. Specifically, the orthographic information of L2 was represented at 139 ms post-stimulus onset in the prefrontal region. At 170 ms post-stimulus onset, the orthographic information of L2 was represented in the occipital region (175–307 ms) and extended to the central parietal and bilateral temporoparietal regions (260–330 ms). At 400 ms post-stimulus onset (380–495 ms), the orthographic information of L2 was represented in almost the whole brain. The phonological information of L2 was represented beginning from 142 to 195 ms in the prefrontal region. At 200 ms post-stimulus onset (195–320 ms), the phonological information of L2 was represented in the occipital region and extended to the right temporoparietal region. At 400 ms post-stimulus onset (365–490 ms), the phonological information was represented in almost the whole brain. The neural representation of lexical information in L1 was weaker in the lexical decision task. Specifically, the orthographic information of L1 was represented between 377 and 432 ms in the middle prefrontal and parietal regions and between 387 and 410 ms in the right prefrontal and temporoparietal regions. The phonological information of L1 was represented only between 450 and 475 ms in the left prefrontal and temporoparietal regions.

In the semantic judgment task, the pattern of lexical information representation in L1 and L2 was opposite to that observed in the lexical decision task. The lexical information of L1 was represented extensively throughout the entire brain, while the information representation of L2 was weaker. Specifically, the orthographic information of L1 was represented at 170 ms in almost the whole brain (159–276 ms) except in the right prefrontal subregions and was later sustained at 395–472 ms across all regions. The phonological representation of L1 began at 129–194 ms in the middle and right occipital regions and extended to the right temporoparietal region at 140–194 ms. At 434–450 ms, the phonological information of L1 was represented in the parietal and the right temporoparietal regions. The neural representation of L2 orthographic information began at 170 ms post-stimulus onset (171–238 ms) in the parietal and bilateral temporoparietal regions and extended to the left prefrontal (214–230 ms) and middle occipital regions (197–238 ms). At 321–369 ms, the orthographic information of L2 was represented in the left and middle occipital regions. The phonological information of L2 was represented only at 200 ms post-stimulus onset (184–220 ms) in the parietal and bilateral temporoparietal regions.

To avoid potential confounding effects of semantic information, we incorporated the semantic similarity as a covariate to further validate the results of the lexical information representation (Supporting Information Figure S2). Results showed that the pattern of pRSA results remained qualitatively identical to that of the original RSA analyses (i.e., without controlling for semantic similarity).

Taking advantage of EEG and RSA, Experiment 2 explored the spatiotemporal representation of lexical information in two languages and the modulatory effects of processing demands. Echoing previous literature (Kriegeskorte et al., 2008; Lo et al., 2019; Mahé et al., 2015), both tasks evoked classical ERP components of word reading, such as the N170, P200, and N400, which have been consistently identified as the crucial component in the processing of orthographic (Kim & Lai, 2012; Lo et al., 2019), phonological (Kong et al., 2010; Lee et al., 2022), and semantic information in the visual word (Antúnez et al., 2022; Delaney-Busch et al., 2019). These findings indicated that the participants completed tasks conscientiously. More importantly, evidence of spatiotemporal RSA revealed that the lexical information of both languages was activated in the brain for Chinese–English bilinguals during L2 word reading. This finding further confirmed the findings obtained by fMRI technology in Experiment 1, providing direct evidence for the hypothesis of bilingual non-selective access from a temporal perspective. More importantly, we found that the processing demands modulated the pathway of lexical access in both languages for bilinguals. Specifically, L2 exhibited statistically significant neural representations of lexical information across a broader time window when bilinguals focused more on the processing of lexical information in L2 (i.e., the lexical decision task), whereas L1 exhibited statistically significant neural representations of lexical information across a broader time window when bilinguals focused more on the processing of semantic information in L2 (i.e., the semantic judgment task).

## DISCUSSION

The present study combined high spatial resolution fMRI (Experiment 1) and high temporal resolution EEG (Experiment 2), and employed RSA to systematically explore the lexical representations of two languages in Chinese–English bilinguals and the modulatory effects of processing demands. The results of Experiment 1 demonstrated that lexical information of L1 (Chinese) was represented in brain regions associated with lexical reading (e.g., IFG, aSMG, and AG) during L2 (English) word reading in Chinese–English bilinguals, and the neural representation of such lexical information was modulated by processing demands. More importantly, spatiotemporal RSA results in Experiment 2 further revealed that lexical information in L1 was activated simultaneously when the L2 words were processed. In addition, the processing demands modulated the spatiotemporal neural representation of lexical information in both languages of bilinguals. These findings indicate that even bilinguals with different language systems (i.e., logographic language such as Chinese and alphabetic language such as English) still follow the non-selective processing mechanism, providing direct evidence for the bilingual non-selective access hypothesis from the perspective of the spatiotemporal combination.

The results of our study made three significant contributions to the literature on the neural representation of bilingual word reading. Firstly, we clarified the neural representation of lexical information in two languages of bilinguals by high spatial resolution fMRI and RSA. Prior research has explored the neural activity of bilingual word reading by fMRI technology and univariate activation analysis, which could only conduct qualitative investigations and lose the fine-grained pattern information (Gu et al., 2023; Lu et al., 2021). The present study used ROI-based and whole-brain-based RSA to explore the neural representation of lexical information from a quantitative perspective. Results indicated that both the PO and PT subregions of the left IFG play an important role in bilingual word reading for both tasks. The PO and PT subregions of the left IFG have been consistently identified as a crucial brain area of multifunctional intersection, such as early word recognition (Woodhead et al., 2014), phonological processing (Buchweitz et al., 2012; Murphy et al., 2019), and semantic integration (Huang et al., 2024; Li et al., 2023). In addition, this area has also been found to exhibit important roles in cognitive control (Diveica et al., 2023; Fu et al., 2017). Thus, it is reasonable to infer that the left IFG may be responsible for phonological representation and information control in the other language when bilinguals process one language.

The second contribution is to further investigate the spatiotemporal representation of lexical information in two languages of bilinguals by high temporal resolution EEG and RSA. Specifically, for both tasks, lexical information of L2 was represented within a 170–400 ms time window, which involved most areas of the whole brain, and lexical information of L1 was represented nearly 400 ms in frontal and parietal regions. Previous studies have consistently suggested that P200 is an essential component of Chinese orthographic processing and may also be related to the phonological processing of Chinese (Liu et al., 2003; Tong et al., 2020). Consequently, we infer that the lexical information of Chinese may undergo orthographic processing and orthography-to-phonology mappings around 200 ms after the presentation of the L2 word. In addition, lexical information of two languages was represented around 400 ms in most areas of the whole brain. Previous studies have reported that the processing of the semantic information began at 200 ms and was later sustained at the N400 and P600 components (Amsel et al., 2013; Kutas & Federmeier, 2011; Zou et al., 2020). Consequently, we infer that the lexical information of two languages may undergo the interaction of lexical and semantic information around 400 ms. These results provide further support for the bilinguals’ non-selective access hypothesis.

The third contribution of this study is to elucidate the modulating effects of processing demands on the spatiotemporal neural representation of lexical information in both languages for bilinguals. Specifically, the RSA results of Experiment 1 showed that when the processing of L2 required more lexical information, the lexical information of L2 words was represented in more widely brain regions. In contrast, when the processing of L2 required more semantic information, the lexical information in L1 was represented in more widely brain regions. We attributed this result to two reasons. First, it may derive from differential processing demands: the lexical decision task involves shallow lexical processing, rendering the non-target language (L1) representation undetectable (Baek et al., 2023; Coltheart et al., 2001; Krause et al., 2024). In contrast, the semantic judgment task primarily engages post-lexical processing with deeper cognitive demands, which consequently results in a robust amplification of the non-target language (L1) representation (Hsiao et al., 2020; Li et al., 2023). Second, it may be attributed to the reliance on L1 mediation during the semantic processing of L2 in an unbalanced bilingual (Kroll & Stewart, 1994; Kroll et al., 2010). Specifically, the lexical information of L2 could be accessed directly, without the need to access the lexical information of L1. However, given that the participants in the present study were unbalanced bilinguals, successful completion of the semantic judgment task required them to mediate L2 processing via L1—that is, to translate L2 words into their L1 equivalents to access stable and precise semantic representations (Deng et al., 2025; Potter et al., 1984). Consequently, the semantic judgment task entailed a greater involvement of L1 across a broader cortical network compared with the lexical decision task. Similarly, the modulating effects of processing demands were further demonstrated on the temporal dynamics of lexical access in Experiment 2, which was consistent with previous studies (Huang et al., 2023; Wang & Maurer, 2017). Specifically, for the lexical decision task, lexical information of L2 was represented across a broader expanse of brain regions within 100–500 ms, whereas lexical information of L1 was solely represented in the prefrontal and frontal-parietal regions at approximately 400 ms. However, for the semantic judgment task, lexical information of L2 was represented in certain brain regions in the left hemisphere within 170–400 ms, while lexical information of L1 was represented in a wider range of brain regions within 150–450 ms. These results were inconsistent with the BIA+ hypothesis, which assumed that lexical recognition itself was not modulated by the task decision system (Dijkstra & van Heuven, 2002). Specifically, the BIA+ hypothesis proposes that the lexical recognition system is directly influenced only by linguistic factors such as lexical, semantic, and syntactic information, rather than non-linguistic factors such as processing demands (van Hell & Dijkstra, 2002). However, the results of our study oppose this assumption of the BIA+ hypothesis. Specifically, our results showed that under different processing tasks, bilinguals exhibit more pronounced differences in the early stages of lexical access of their two languages, indicating that processing demands affect not only the subsequent judgment of task decisions but also the early lexical recognition stage of information processing.

Notably, the lexical information of L1 was barely represented under the lexical decision task. While the lexical information of two languages was consistently detected in both the fMRI study and the EEG study. These representational results jointly indicate that the bilinguals’ non-selective access hypothesis occurs only under conditions of high semantic processing demands.

Two limitations should be mentioned. First, participants in our study were unbalanced bilinguals. Previous studies have found that increased L2 proficiency enhanced the similarity between two languages (Li et al., 2019; Xu et al., 2021). Therefore, future research needs to recruit highly proficient L2 bilinguals to further validate the conclusions. Second, the two tasks used in this study had inconsistent repetition of probe stimuli (1 time per probe in the lexical decision tasks, whereas 3 times per probe in the semantic judgment tasks) and key-press requirements (pressing the button for each trial in the lexical decision task, whereas pressing the button only for the color word in the semantic judgment task). The neural activity induced by the discrepancy in the repetition of probe stimuli and key-press responses may confound the neural activity associated with reading demands. Therefore, future research needs to employ different reading tasks with the same number of probes and consistent response requirements to precisely explore the modulatory effects of processing demands. Third, due to the relatively obvious grapheme-to-phoneme correspondence (GPC) rules in alphabetic language (Fischer-Baum et al., 2017; Gu et al., 2026), the inherent correlation between orthographic and phonological RDMs may introduce partial confound into the interpretation of the neural representation of the two languages in bilinguals. Therefore, future research needs to employ stimuli that deviate from the GPC rules (e.g., irregular words) as experimental materials to disentangle the contributions of orthography and phonology to the neural representation of the two languages in bilinguals.

In conclusion, the present study revealed that processing demands modulate the neural representation of the two languages in bilinguals. More importantly, during reading tasks with high semantic demands, even bilinguals with different language systems (i.e., logographic languages and alphabetic languages) still follow the non-selective processing mechanism. Our findings provide direct neuroimaging evidence for the hypothesis of bilingual non-selective access from the perspective of the spatiotemporal combination, indicating that non-selective processing in bilinguals occurs only under conditions of high semantic processing demands.

## Acknowledgments

This study was supported by grants from the Guangdong Basic and Applied Basic Research Foundation [2024A1515011023], the National Natural Science Foundation of China [32271098, 32571226], the Research Center for Brain Cognition and Human Development, Guangdong, China [2024B0303390003], and the Striving for the First-Class, Improving Weak Links and Highlighting Features (SIH) Key Discipline for Psychology in South China Normal University.

## Funding Information

Leilei Mei, Basic and Applied Basic Research Foundation of Guangdong Province (https://dx.doi.org/10.13039/501100021171), Award ID: 2024A1515011023. Leilei Mei, National Natural Science Foundation of China (https://dx.doi.org/10.13039/501100001809), Award ID:32271098. Leilei Mei, National Natural Science Foundation of China (https://dx.doi.org/10.13039/501100001809), Award ID: 32571226. Leilei Mei, Research Center for Brain Cognition and Human Development, Guangdong, Award ID: 2024B0303390003. Leilei Mei, Striving for the First-Class, Improving Weak Links and Highlighting Features (SIH) Key Discipline for Psychology in South China Normal University.

## Author Contributions

**Xiaoyu Liu**: Conceptualization: Equal; Data curation: Equal; Formal analysis: Equal; Methodology: Equal; Visualization: Equal; Writing – original draft: Equal; Writing – review & editing: Lead. **Lala Gu**: Conceptualization: Equal; Data curation: Equal; Formal analysis: Equal; Methodology: Equal; Visualization: Equal; Writing – original draft: Equal; Writing – review & editing: Lead. **Xiaoxue Feng**: Visualization: Supporting; Writing – original draft: Supporting. **Yuan Feng**: Visualization: Supporting; Writing – original draft: Supporting. **Xingying Lin**: Writing – review & editing: Supporting. **Nannan Gu**: Writing – review & editing: Supporting. **Leilei Mei**: Conceptualization: Lead; Methodology: Lead; Visualization: Lead; Writing – original draft: Lead; Writing – review & editing: Lead.

## DATA AND CODE AVAILABILITY STATEMENTS

The materials, statistical code, and data are available at OSF (https://osf.io/uxhqp/).

## Supplementary Material



## TECHNICAL TERMS

RSA: Representational similarity analysis.
BIA+ model: The Bilingual Interactive Activation + model.
PO: Pars opercularis.
PT: Pars triangularis.
aSMG: Anterior superior marginal gyrus.
AG: Angular gyrus.
pSTG: Posterior superior temporal gyrus.
pMTG: Posterior middle temporal gyrus.
pITG: Posterior inferior temporal gyrus.
FG: Fusiform gyrus.
