# Supplementary material for: Lexical Representations of the Native and Second Languages During L2 Word Reading in Chinese–English Bilinguals
Source: Neurobiol Lang (Camb). 2026 Jun 16;7:NOL.a.255. doi: 10.1162/NOL.a.255 (PMC13293728; doi:10.1162/NOL.a.255)

Table S1 The ROIs identified based on the Harvard-Oxford probabilistic atlas.

**ROI**	**Harvard-Oxford probabilistic atlas**
PO	Inferior Frontal Gyrus, pars opercularis (6)
PT	Inferior Frontal Gyrus, pars triangularis (5)
AG	Angular Gyrus (21)
aSMG	Supramarginal Gyrus, anterior division (19)
pSTG	Superior Temporal Gyrus, posterior division (10)
pMTG	Middle Temporal Gyrus, posterior division (12)
pITG	Inferior Temporal Gyrus, posterior division (15)
FG	Temporal Occipital Fusiform Cortex (39)

Table S2 The 9 subregions in Experiment 2 and the electrodes they contain.

Subregion	Electrodes
1	F3、F5、F7、FC3、FC5、FT7
2	F1、Fz、F2、FC1、FCz、FC2
3	F4、F6、F8、FC4、FC6、FT8
4	C3、C5、CP3、CP5、TP7
5	C1、Cz、C2、CP1、CPz、CP2
6	C4、C6、CP4、CP6、TP8
7	P3、P5、P7、PO5、PO7、O1
8	P1、Pz、P3、PO3、POz、PO4
9	P4、P6、P8、PO6、PO8、O2

Fig S1 The classical ERP components evoked in the lexical decision and the semantic judgment tasks.

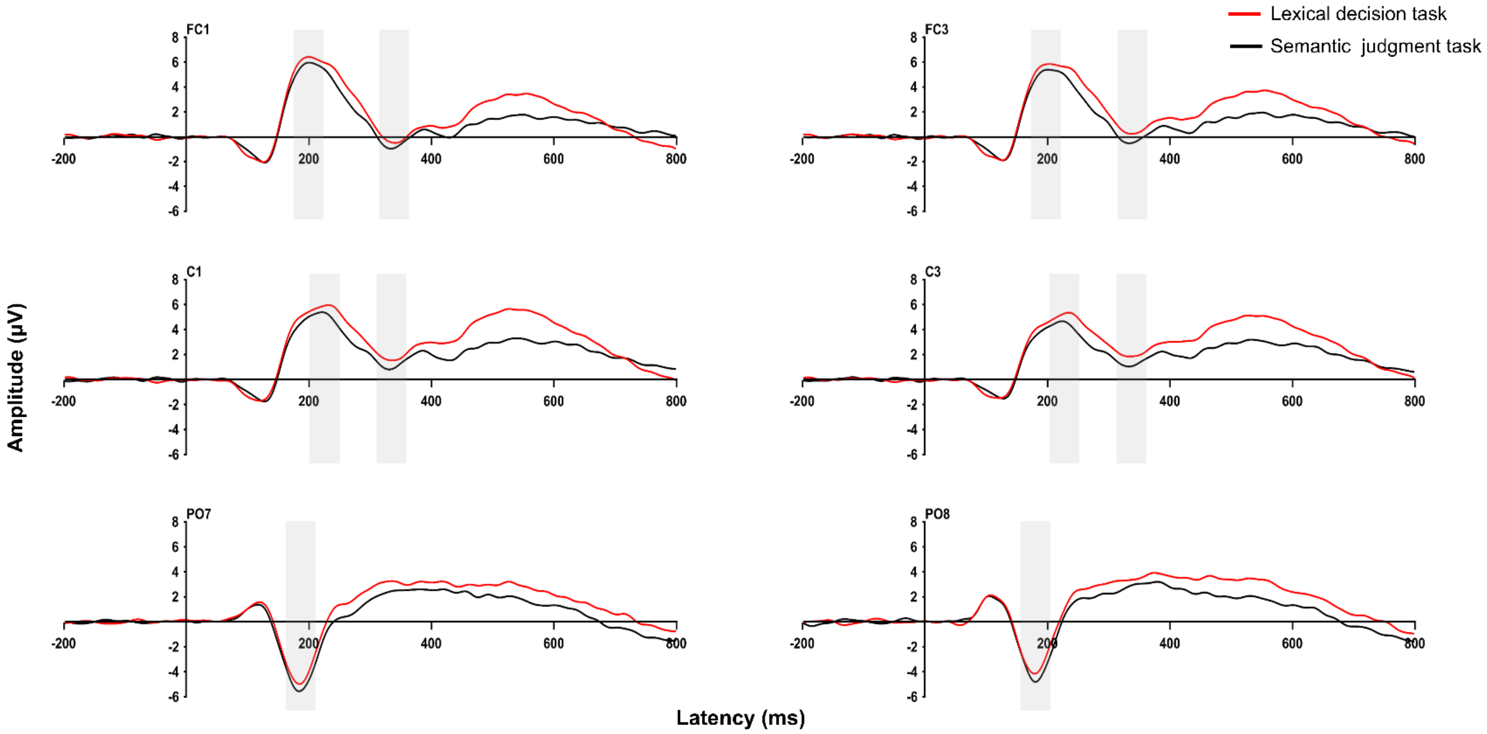

Fig S2 Spatiotemporal RSA results with (A) and without (B) controlling for semantic similarity in the lexical decision (the left panel) and the semantic judgment (the right panel) task. The x-axis displays the post-stimulus time course (0-600 ms). The y-axis indicates the 9 subregions. Blue clusters indicate significant time points identified by the cluster-corrected permutation test.

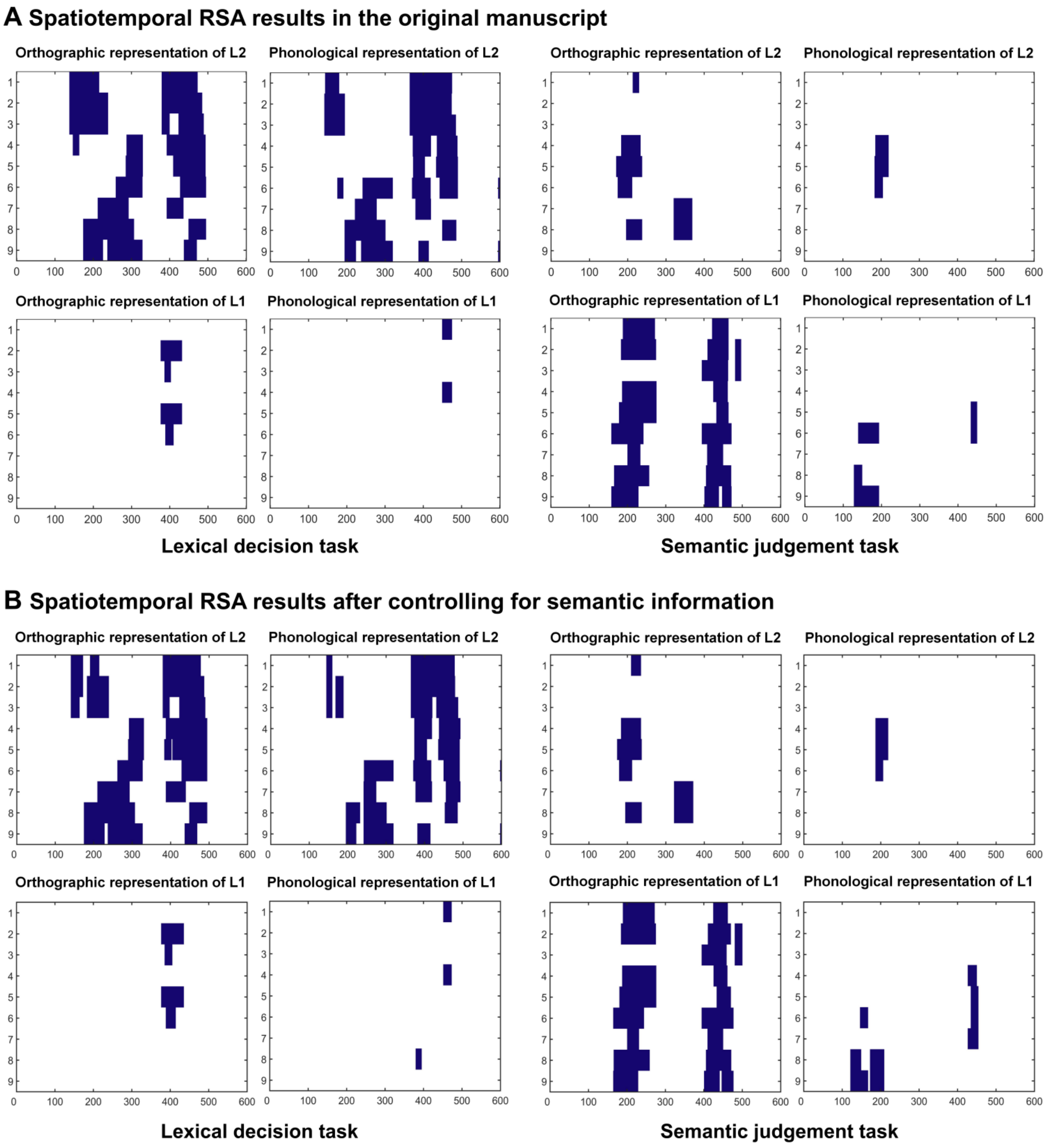

Supplement: Supplementary file 1 [file nol-07-255-s001.pdf]
